# Patient experiences with outpatient care in Hungary: results of an online population survey

**DOI:** 10.1007/s10198-019-01064-z

**Published:** 2019-05-16

**Authors:** Óscar Brito Fernandes, Petra Baji, Dionne Kringos, Niek Klazinga, László Gulácsi, Armin Lucevic, Imre Boncz, Márta Péntek

**Affiliations:** 1 Department of Health Economics, Corvinus University of Budapest, Fővám tér 8., Budapest, 1093 Hungary; 2 Department of Public Health, Amsterdam Public Health Research Institute, Amsterdam UMC, University of Amsterdam, Meibergdreef 9, 1105 AZ Amsterdam, The Netherlands; 3 Institute for Health Insurance, Faculty of Health Sciences, University of Pécs, Mária u. 5-7, Pécs, 7621 Hungary

**Keywords:** Patient-reported experience measure, Quality of care, Outpatient care, OECD, Survey, Hungary, C83, I12, I18

## Abstract

**Background:**

Health systems are undertaking efforts to make health care more patient centered and value based. To achieve this goal, the use of patient-reported experience measures (PREMs) is increasing, especially across OECD countries. However, in Hungary, data on patients’ experiences are still lacking. Thus, our aim was twofold: first, to collect data on outpatient experience in Hungary on patient–doctor communication and patient involvement in decision making and compare it with that of other OECD countries; second, to assess associations of outpatient experience with patients’ socioeconomic characteristics.

**Methods:**

In early 2019, we conducted a cross-sectional, online, self-administered survey in a national representative sample of Hungary’s population (*n* = 1000). The sample was weighted considering gender, age, highest education level attained, type of settlement, and region of residence. The survey questions were based on a set of recommended questions by the OECD.

**Results:**

Our findings show that the proportion of reported positive experiences is as follows: doctors providing easy-to-understand explanations (93.1%) followed by time spent on the consultation (87.5%), opportunities to raise questions (85.8%), and doctors involving patients in decision making about care and treatment (80.1%). The share of positive experiences falls behind OECD’s average regarding patient–doctor communication and patient involvement in decision making, which signals room for improvement in these areas.

**Conclusions:**

Women, younger people, people with a paid job, and patients with consultations with allied health professionals reported significant lesser positive care experiences and, hence, more targeted policies can be initiated based on our findings.

**Electronic supplementary material:**

The online version of this article (10.1007/s10198-019-01064-z) contains supplementary material, which is available to authorized users.

## Introduction

Health systems are committed to redesigning the delivery of care in an endeavor to make health care more patient centered and value based. To achieve this goal, health systems seek to understand and act upon on how patients perceive and experience their trajectories of care. Through the more active engagement of patients in this, one may expect health systems to become more responsive to population’s needs and expectations on non-medical and non-financial aspects of the care process, resulting in better quality of care [1, 2].

To assess patients’ experiences, various patient-reported experience measures (PREMs) have been developed. PREMs are a multidimensional concept [3] with varying definitions in the literature [4]. These definitions anchor patient experiences measurement as an assessment of the patients’ perception on how their care is provided. Hence, PREMs comprise dimensions of care that are most important to patients: (1) personal interactions and communications with health professionals, (2) autonomy to be involved in decision making about care and treatment, (3) access to care, (4) continuity and coordination of care [5, 6].

Studies have shown associations between patient experiences, the process of care, and outcomes. Two systematic reviews have highlighted that more positive experiences were associated with a decrease of primary and secondary care use (e.g., hospital admissions, readmissions, and primary care consultations), and greater adherence to prevention and treatment processes [7, 8]. Also, evidence shows that inadequate patient–doctor communication and a lesser autonomy of patients in decision making affected clinical effectiveness and safety [9].

Purposes of PREMs use vary across health systems. PREMs have been used for quality accreditation and certification [10], payment programs [4], and to develop health policies that promote patient-centered care [11]. Hence, patient experiences’ measurement and monitoring can highlight the need for changes in health systems to improve quality of care [12].

Many countries of the Organisation for Economic Co-operation and Development (OECD) have developed patient-reported instruments to collect data about patient experiences in different health care settings [13, 14]. Most often these instruments follow previous efforts to measure patients’ experiences, such as those of the Picker Institute, the Agency for Healthcare Research and Quality, and the Commonwealth Fund. The Commonwealth Fund has developed an international health policy survey that measures patient experiences and allows for cross-national comparisons [15].

The use of PREMs has increased among OECD countries, but some are lacking behind such as Hungary. Hungarian governmental authorities have been employing a series of efforts to measure and report health system performance indicators [16–18], but national representative data collection on patients’ experiences are not yet implemented. This may undermine efforts to identify key priority areas for which improvement is needed to enhance Hungary’s health system responsiveness. For example, Hungary exhibits a larger number of outpatient contacts per person/year (11.95), in contrast to that of the EU27 (6.20) and that of the European region (7.85) [19]. However, to our knowledge, no study has yet measured what are patients’ experiences with outpatient care in Hungary. As this information may be of valuable relevance for Hungary’s health system improvement, with this study we seek to fill that gap.

Our aim is twofold: first, to collect data on outpatient experience in Hungary on patient–doctor communication and patient involvement in decision making and compare it with that of other OECD countries, with a closer look at the Central and Eastern European members; second, to assess associations of outpatient experience with patients’ socioeconomic characteristics.

## Methods

### Study design and population

A cross-sectional, online, self-administered survey was conducted in early 2019 in Hungary. Respondents from the general adult population (*n* = 1000) were recruited in early 2019 from a large online panel of a survey company (Big Data Scientist Kft.). A quota sampling approach was employed to ensure a representative sample for Hungary’s population in terms of gender, age, highest education level attained, type of settlement, and region of residence. The process aimed at having *n* = 1000 as a target sample size. This study received an ethical approval from the Medical Research Council of Hungary (Nr. 47654-2/2018/EKU).

### Survey

The survey ‘Patient experiences in healthcare’ consisted of three modules of questions (‘eHealth Literacy’, ‘Shared Decision Making’ and ‘Patient Reported Experience Measures’). The ‘Patient Reported Experience Measures’ module focused on patient experiences with outpatient care.

Questions to assess patients' experience with outpatient care were based on a previously published set of recommended questions by the OECD [10]. A Hungarian version of the questions was developed in the following way: first, a forward–backward translation of the questions was performed; second, we conducted a pre-testing and cognitive interviewing, involving one interviewer and five respondents. In this pre-test, respondents were asked to complete the questionnaire in the presence of the interviewer and were able to interrupt the questionnaire to raise questions. After that, the interviewer and respondent read through the questions and discussed each statement. We adapted the answer options for the question on type of care received to match the Hungarian context and culture. The rest of the questions were straightforward, with no culturally sensitive wording; thus, we were able to keep the wording as exactly to the original as possible.

### Variables

#### Dependent variables

Survey participants were asked to answer questions with regard to their last consultation/examination in the previous 12 months. If the respondent visited outpatient care during the last 12 months, we asked about: (1) the doctor spending enough time with the patient in consultation; (2) the doctor providing easy to understand explanations; (3) the doctor giving the opportunity to ask questions or raise concerns; (4) the doctor involving the patient in decisions about care and treatment. The questions had a four-point Likert scale response option (yes, definitely; yes, to some extent; no, not really; no, definitely not) that was presented in Hungarian language as: *igen, egyértelműen igen*; *igen, bizonyos mértékig*; *nem, nem igazán*; *nem, egyértelműen nem*. Additionally, the respondent was able to answer, “I do not know” (*nem tudom*) or “I do not want to answer” (*nem kívánok válaszolni*) to all questions. Respondents had two more response options in the last question: “No, I did not want to be involved” (*nem, nem akartam, hogy bevonjon a döntésbe*) and “Not applicable: no decisions about treatment were made” (*nem alkalmazható: nem történt kezeléssel kapcsolatos döntés*). Answers were later dichotomized into Yes (1: positive experience, combining responses *Yes, definitely* and *Yes, to some extent*) and No (0: did not occur, combining responses *No, definitely not* and *No, not really*) for further analysis. Computed proportions of patients’ experiences omit the “I do not know” or “I do not want to answer” answers. For the purpose of this research, we analyzed data from a subsample of respondents that received outpatient care within the last 12 months.

#### Independent variables

To explore associations of outpatient experience with patients’ socioeconomic characteristics, we included in the analysis the following variables: gender (men/women), age group (18–24; 25–34; 35–44; 45–54; 55–64; 65+); highest education attained (primary or less; secondary; tertiary); net household income per capita (5 quintiles); self-reported health status (excellent; very good; good; fair; poor); if suffering from a chronic illness lasting (or expected to last) at least 6 months (1: yes; 0: no); marital status (1: married or similar; 0: single); medical qualification (1: holds a medical qualification; 0: no medical qualification); employment status (1: having a paid job; 0: not having a paid job); type of settlement (urban: Budapest; other towns; and rural area: village); region of residence (Central Hungary; Eastern Hungary; Western Hungary); and type of consultation (general practitioner/family physician; specialist doctor at an outpatient public facility; specialist doctor at an outpatient private facility; allied health professional at an outpatient public facility; allied health professional at an outpatient private facility; telephone consultation).

### Statistical analysis

Survey sample was weighted considering gender, age, highest education level attained, type of settlement, and region of residence. Weights accounted for the nominal distribution across the adult population in Hungary (based on the latest census data from 2011) and were taken into account in all statistical analyses.

Characterization of the sample included absolute and relative frequency to all independent variables, considering both the unweighted sample and the computed weights. Independence of each factor (independent variable) to the four dependent variables was assessed. Candidate factors for modeling included all variables associated with, at least, one of the dependent variables at *p* < 0.20, as determined by bivariate analyses. We used Pearson’s $\chi^{2}$ statistic for all bivariate analyses of categorical data. To account for the complex sample survey design (due to sample weights), this statistic was turned into an *F* statistic.

We performed logistic regression for multivariate analysis to identify which factors were associated with positive experiences in outpatient care. To achieve convergence in all multivariate models, with better fitting to data, respondents reported to receive care by telephone appointment (weighted proportion of 0.6%) were excluded from the analysis.

All analyses were performed using Stata version 14, using the survey commands. Confidence level was set at 95%.

## Results

A total of 1000 questionnaires were completed. Sample was weighted to account for differential sampling probabilities and reflect the distribution of Hungary’s 2011 census (Table 1). Women were more represented (53.4%), 53.7% of the sample was 45 years and over, and 51% reported having only primary education or less. The sample captured an evenly distribution of respondents across Hungary: Central (30%), Eastern (39.6%) and Western (30.4%).

**Table 1:** Sample’s socioeconomic characteristics

	Weighted		Unweighted	
	Population size	Proportion (%)	*N*	%
Gender	**1000**		**1000**	
Women		53.4	550	55.0
Men		46.6	450	45.0
Age groups (years)	**1000**		**1000**	
18–24		10.6	118	11.8
25–34		16.9	198	19.8
35–44		18.8	191	19.1
45–54		15.5	125	12.5
55–64		17.6	147	14.7
65+		20.6	221	22.1
Highest education completed	**1000**		**1000**	
Primary or less		51.0	341	34.1
Secondary		31.3	363	36.3
Tertiary		17.7	296	29.6
Net household income per capita	**839**		**822**	
Quintile 1		28.2	195	23.7
Quintile 2		18.8	143	17.4
Quintile 3		20.0	165	20.1
Quintile 4		19.2	174	21.2
Quintile 5		13.9	145	17.6
Self-reported health status	**1000**		**1000**	
Excellent		7.3	81	8.1
Very good		26.9	283	28.3
Good		38.4	400	40.0
Fair		23.0	205	20.5
Poor		4.4	31	3.1
Employment status	**1000**		**1000**	
Not having a paid job		51.5	500	50.0
Having a paid job		48.5	500	50.0
Type of settlement	**1000**		**1000**	
Budapest		18.1	213	21.3
Other towns		51.9	557	55.7
Village		30.0	230	23.0
Region	**1000**		**1000**	
Central Hungary		30.0	348	34.8
Eastern Hungary		39.6	353	35.3
Western Hungary		30.4	299	29.9
Type of consultation	**725**		**736**	
General practitioner/family physician		42.0	305	41.4
Specialist at an outpatient public facility		43.2	311	42.3
Specialist at an outpatient private facility		8.8	79	10.7
Allied health professional at an outpatient public facility		4.0	26	3.5
Allied health professional at an outpatient private facility		1.4	10	1.4
Telephone consultation		0.6	5	0.7

Primary level of education included those who had fully completed primary education and who partly completed secondary education without direct access to post-secondary or tertiary education. Secondary level of education included those who fully completed secondary education or attended tertiary education without completing it. Tertiary level of education included those who had fully completed university studies

Net household income per capita (in thousands of Hungarian forints): Quintile 1 < 65; 65 < Quintile 2 < 91.5; 91.5 ≤ Quintile 3 < 130; 130 ≤ Quintile 4 < 185; Quintile 5 ≥ 185

Difference in the unweighted sample size for net household income per capita is because 3% of respondents (*n* = 30) were not sure of their monthly household net income, and 14.8% (*n* = 148) declined to answer

Difference in the unweighted sample size for type of consultation is because 19.6% of respondents (*n* = 196) had a consultation more than 12 months ago, 4.4% (*n* = 44) were not sure of an answer, and 2.4% (*n* = 24) declined to answer

Employment status: “Not having a paid job” category included people retired, with a disability pension, university students, unemployed looking for a job, unemployed not looking for a job, housewife, and other; “having a paid job” category included those working full-time or part-time

The population size on the weighted column account for the complex survey design computed weights

### Experiences with outpatient care and comparison with OECD countries

Our analysis focused on respondents that reported having a medical appointment or examination within the last 12 months prior to this study, which accounted for 72.5% of the full sample. Of this subsample, most respondents had an appointment with a general practitioner/family doctor (GP) (42%) or with a specialist doctor at an outpatient public facility (43.2%). Consultations by telephone (< 1%) or with an allied health professional (< 4%) were not frequent.

**Fig. 1:**
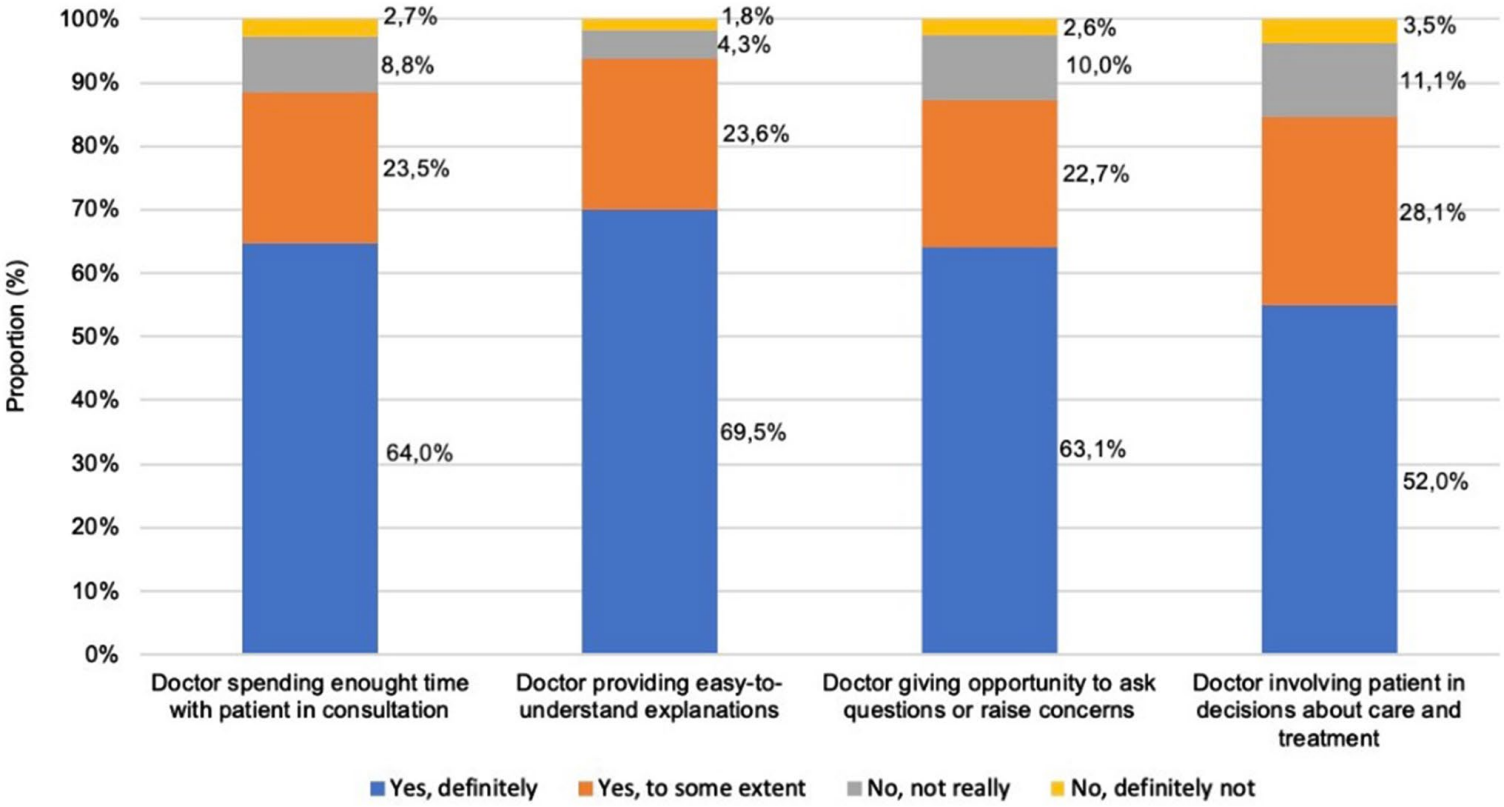
Proportions of reported positive experiences with outpatient care in Hungary

The proportion of respondents that reported positive experiences is as shown in Fig. 1, with proportions omitted whether respondents declined to answer (0.1–0.3%) or were not sure of an answer (0.7–1.3%).

Overall, 87.5% of respondents reported that a doctor spent enough time with them during consultation (Yes, definitely/Yes, to some extent) (Fig. 1). In contrast to the OECD18 average, Hungary has a higher proportion of respondents that perceived the duration of the consultation as adequate (Fig. S1, Supplementary material). Moreover, the share of positive experiences in Hungary is similar to that of the UK, France, and Estonia, larger than that of Poland, but behind that of the Czech Republic.

In total, 93.1% reported that a doctor provided easy to understand explanations. This share of positive experiences is aligned with that of the OECD18 average, New Zealand, or Korea. In addition, it is higher than that reported by France, Germany, Estonia, or Poland (Fig. S2, Supplementary material).

More than 85% of the respondents reported having been given the opportunity to raise questions at the consultation. This share of positive experiences is similar to that of the OECD19 average and that of France or Estonia, whereas higher than that reported by Sweden, Japan, or Poland (Fig. S3, Supplementary material).

A total share of 80.1% of respondents were involved by a doctor in decision making about their care and treatment. Countries with a similar proportion were France, Israel, Norway, and Sweden. However, Hungary has a higher proportion of patients that feel involved in decision making than that of Spain, Poland, or Estonia (Fig. S4, Supplementary material).

### Associations of outpatient experience with patients’ socioeconomic characteristics

#### Time of consultation

Proportion of respondents that believed that a doctor spent enough time at the consultation was significantly higher among men (92.5% vs 84.7%), people without a paid job (91.1% vs 85.3%), and increased with age (e.g., respondents 65 years and older: 95.6% vs 25–34 category: 78.4%) (Table 2). Proportion of positive experiences varied significantly across types of consultation. The majority of the responders reported positive experiences with the GPs (89.2%). Consultations with allied health professionals, whether at an outpatient public facility (66.7%) or at a private facility (59.5%), presented lesser proportions of positive experiences compared to those of specialist doctors at an outpatient public facility (91%) or at a private facility (87.2%).

**Table 2:** Proportion of positive experiences with outpatient care by sample’s characteristics (weighted population size = 725)

	Doctor spending enough time with patient in consultation		Doctor providing easy to understand explanations		Doctor giving opportunity to ask questions or raise concerns		Doctor involving patient in decisions about care and treatment	
	Yes (%)	*F* (*p* value)	Yes (%)	*F* (*p* value)	Yes (%)	*F* (*p* value)	Yes (%)	*F* (*p* value)
Gender								
Women	84.7	8.859 (**0.003**)	92.9	1.363 (0.243)	84.9	3.190 (0.075)	81.2	5.896 (**0.015)**
Men	92.5		95.1		89.8		88.4	
Age groups (years)								
18–24	80.9	4.065 (**0.001**)	89.7	1.409 (0.218)	81.0	2.542 (**0.028**)	75.1	2.508 (**0.029)**
25–34	78.4		90.4		79.3		76.9	
35–44	86.3		93.3		87.3		89.4	
45–54	88.4		93.2		85.1		83.3	
55–64	92.2		96.2		90.1		84.8	
65+	95.6		96.8		93.3		89.9	
Highest education completed								
Primary or less	86.5	1.425 (0.2412)	92.5	1.778 (0.171)	84.7	2.676 (0.073)	81.9	2.223 (0.111)
Secondary	89.6		96.1		88.1		88.1	
Tertiary	91.1		93.9		91.8		85.6	
Net household income per capita								
Quintile 1	82.9	1.217 (0.302)	92.7	0.130 (0.970)	85.7	0.354 (0.84)	81.6	1.046 (0.382)
Quintile 2	88.7		94.2		88.4		90.1	
Quintile 3	88.1		94.6		85.5		83.6	
Quintile 4	89.4		94.4		89.9		85.5	
Quintile 5	92.1		93.4		88.2		80.0	
Self-reported health status								
Excellent	91.4	0.520 (0.709)	94.0	2.770 (**0.027**)	90.5	1.669 (0.156)	84.6	1.397 (0.235)
Very good	85.9		90.4		85.1		82.5	
Good	90.0		97.6		91.1		88.8	
Fair	87.1		91.6		83.0		80.8	
Poor	91.1		94.3		85.8		81.8	
Employment status								
Not having a paid job	91.1	4.934 (**0.027**)	94.8	0.912 (0.340)	90.5	6.616 (**0.01**)	86.1	1.183 (0.277)
Having a paid job	85.3		93.0		83.5		82.9	
Type of settlement								
Budapest	87.7	0.113 (0.888)	95.7	0.711 (0.481)	90.0	0.521 (0.588)	85.9	1.002 (0.366)
Other towns	89.0		93.0		86.7		82.6	
Village	87.7		94.6		86.3		87.3	
Region								
Central Hungary	89.6	0.372 (0.688)	95.5	0.918 (0.397)	89.6	1.552 (0.212)	86.7	1.109 (0.330)
Eastern Hungary	87.0		92.5		87.9		85.4	
Western Hungary	88.8		94.1		83.8		81.4	
Type of consultation								
General practitioner/family physician	89.2	4.666 (**< 0.001**)	94.4	4.227 (**< 0.001**)	88.0	1.755 (0.122)	83.7	1.155 (0.329)
Specialist at an outpatient public facility	91.0		95.2		86.7		85.4	
Specialist at an outpatient private facility	87.2		95.3		93.0		90.4	
Allied health professional at an outpatient public facility	66.7		79.2		81.8		78.3	
Allied health professional at an outpatient private facility	59.5		67.2		59.5		60.6	
Telephone consultation	60.0		100.0		84.2		84.2	

*P* values in bold show statistically significant differences (*p* < 0.05)

According to regression analysis (Table 3), men were significantly more likely to experience that a doctor spent enough time in consultation than women [odds ratio (OR): 2.114], while respondents younger than 35 years old were significantly less likely to report positive experiences than respondents above 65. In addition, types of consultation that were least likely to provide a positive experience were those of allied health professionals (OR = 0.163 and 0.156).

**Table 3:** Multivariate logistic regression results to assess determinant characteristics with positive patient experiences with outpatient care (weighted population size = 725)

	Doctor spending enough time with patient in consultation		Doctor providing easy to understand explanations		Doctor giving opportunity to ask questions or raise concerns		Doctor involving patient in decisions about care and treatment	
Covariate	Adjusted OR	(95% CI)	Adjusted OR	(95% CI)	Adjusted OR	(95% CI)	Adjusted OR	(95% CI)
Gender								
Women	(Reference)	–	(Reference)	–	(Reference)	–	(Reference)	–
Men	2.114	(1.065–4.197)	1.344	(0.555–3.259)	1.507	(0.759–2.991)	1.810	(1.029–3.181)
Age groups (years)								
18–24	0.274	(0.077–0.976)	0.231	(0.039–1.372)	0.157	(0.449–0.551)	0.253	(0.099–0.646)
25–34	0.275	(0.085–0.893)	0.287	(0.056–1.466)	0.190	(0.587–0.618)	0.364	(0.143–0.925)
35–44	0.589	(0.173–2.012)	0.603	(0.099–3.664)	0.493	(0.144–1.690)	1.077	(0.399–2.905)
45–54	0.614	(0.182–2.074)	0.433	(0.072–2.612)	0.440	(0.133–1.455)	0.632	(0.234–1.707)
55–64	0.908	(0.306–2.699)	0.707	(0.135–3.712)	0.684	(0.215–2.174)	0.620	(0.260–1.474)
65+	(Reference)	–	(Reference)	–	(Reference)	–	(Reference)	–
Highest education completed								
Primary or less	(Reference)	–	(Reference)	–	(Reference)	–	(Reference)	–
Secondary	1.914	(0.936–3.914)	2.437	(0.990–6.001)	1.946	(1.007–3.760)	2.023	(1.083–3.781)
Tertiary	1.107	(0.508–2.415)	0.985	(0.363–2.672)	2.204	(0.959–5.065)	1.317	(0.660–2.631)
Net household income per capita								
Quintile 1	(Reference)	–	(Reference)	–	(Reference)	–	(Reference)	–
Quintile 2	1.673	(0.726–3.856)	1.278	(0.378–4.317)	1.181	(0.461–3.023)	1.895	(0.828–4.341)
Quintile 3	1.370	(0.545–3.443)	1.159	(0.335–4.009)	0.710	(0.301–1.674)	0.887	(0.419–1.878)
Quintile 4	1.518	(0.598–3.857)	1.185	(0.374–3.758)	1.080	(0.416–2.807)	0.950	(0.427–2.115)
Quintile 5	1.903	(0.695–5.212)	0.756	(0.240–2.382)	0.705	(0.256–1.945)	0.513	(0.212–1.236)
Self-reported health status								
Excellent	(Reference)	–	(Reference)	–	(Reference)	–	(Reference)	–
Very good	0.328	(0.079–1.360)	0.295	(0.068–1.272)	0.290	(0.056–1.487)	0.877	(0.309–2.485)
Good	0.374	(0.089–1.559)	1.062	(0.233–4.833)	0.423	(0.081–2.217)	1.075	(0.387–2.986)
Fair	0.265	(0.057–1.240)	0.372	(0.070–1.975)	0.169	(0.031–0.930)	0.524	(0.174–1.574)
Poor	0.253	(0.032–1.973)	0.332	(0.035–3.132)	0.138	(0.017–1.130)	0.428	(0.093–1.966)
Employment status								
Not having a paid job	(Reference)	–	(Reference)	–	(Reference)	–	(Reference)	–
Having a paid job	0.484	(0.232–1.009)	0.660	(0.257–1.693)	0.467	(0.231–0.944)	0.682	(0.365–1.276)
Type of consultation								
General practitioner/family physician	(Reference)	–	(Reference)	–	(Reference)	–	(Reference)	–
Specialist at an outpatient public facility	1.043	(0.523–2.079)	1.012	(0.400–2.559)	0.725	(0.380–1.383)	1.119	(0.632–1.981)
Specialist at an outpatient private facility	0.850	(0.315–2.297)	1.005	(0.171–5.900)	1.582	(0.392–6.378)	1.872	(0.564–6.219)
Allied health professional at an outpatient public facility	0.163	(0.053–0.501)	0.144	(0.044–0.465)	0.435	(0.121–1.564)	0.608	(0.207–1.791)
Allied health professional at an outpatient private facility	0.156	(0.027–0.888)	0.107	(0.019–0.607)	0.205	(0.041–1.040)	0.396	(0.076–2.070)

OR stands for odds ratio and CI for confidence interval

#### Communication

Older age groups reported higher share of positive experiences on receiving easy to understand explanations (e.g., 96.8% for people 65 years and over, in contrast to 89.7% for people aged 18–24). Consultations with allied health professionals scored lowest proportions of positive experiences on the clarity of explanations (67.2% for consultations in private facilities, and 79.2% in public facilities), whereas consultations with a specialist doctor at an outpatient private facility scored the highest proportion of positive experiences (95.3%). Regression results also showed that respondents that received a consultation with an allied health professional rather than a GP reported significantly fewer positive experiences.

Respondents aged 65 years and over reported more positive experiences on being given the opportunity to ask question compared to those of other age groups (e.g., 93.3% for people 65 years and over, while the proportion for people aged 25–34 was 79.3%). A larger proportion of positive experiences occurred with specialist doctors at private facilities (93%), followed by those of GPs (88%). According to the regression results, respondents with secondary or tertiary education were significantly more likely to perceive they could ask questions or raise concerns (OR = 1.946 and 2.204). Younger respondents (18–24 and 25–34 years old) and people who had a paid job reported significantly lesser positive experiences (OR: 0.157, 0.190, and 0.467). In contrast to a GP consultation, respondents that received private specialist care presented increased likelihood of being given the opportunity to ask questions and raise concerns (OR: 1.582); however, this difference was not significant.

#### Patient involvement in decision making

Women reported significantly fewer involvement in decision making than men (81.2% vs 88.4%). Younger age groups reported significant fewer proportion of positive experiences compared to those of older age groups (e.g., 89.9% of people aged 65 years and over reported to be involved in decisions, in contrast to 76.9% of people aged 25–34). In addition, results suggested that people living in towns (82.6%) or in Western Hungary (81.4%) reported less frequently being involved in decision making.

Regression results showed that men and respondents with secondary education (compared to primary education) were significantly more likely to report being involved in decisions (OR = 2.023). Younger respondents had significant lower odds of reporting a positive experience on whether their doctor involved them in decisions about care and treatment (18–24 age category OR: 0.253; 25–34 age category: 0.364). Respondents with higher net household incomes per capita reported to be less involved in decisions, in contrast to those with the lowest net household income (OR: 0.513); however, this was not significant. Furthermore, people that received care from a specialist doctor faced higher likelihood of being involved in decision making. This effect was larger (but not significant) in private providers (OR: 1.872).

## Discussion

In this study, we assessed outpatient experience in Hungary via a cross-sectional online survey involving a representative sample of the adult population. Explanatory factors were analyzed, and comparisons were made with other OECD countries, with special focus on the Central and Eastern European (CEE) region.

Our findings suggested that the largest proportion of positive experiences was that of a doctor proving easy-to-understand explanations (93.1%), and the least was that of a doctor involving the patient in decision making about care and treatment (80.1%).

In contrast to the OECD average, Hungary had a higher proportion of respondents that perceived time spent on consultation as adequate. However, the share of positive experiences falls behind OECD’s average when it concerns patient–doctor communication and patient involvement in decision making. Compared with other CEE countries, in Hungary, the share of positive experiences was lower than that in the Czech Republic, similar to that in Estonia, but higher than that in Poland. Nevertheless, we need to highlight that some of the OECD data refer to patient experience with general practice/family doctor only, while our study also involved consultations with allied health professional (4% of the sample).

Our international comparison signals room for improvement in communication and shared decision making. One recent study which examined GPs’ perceptions on shared decision making in patients’ referral also found that in Hungary patients’ preferences and interests (costs, traveling and waiting time) were less likely to be taken into account by GPs than in other countries [20]. Nevertheless, studies on this topic are still rare in Hungary; thus, more in-depth research could help to better understand the perceptions, preferences and experiences with shared decision making in Hungary.

Our results also suggest significant differences on outpatient care experiences across patients’ socioeconomic characteristics such as gender, age group, education, employment status, and type of consultation. Overall, men reported a higher proportion of positive experiences, emphasized on timing of consultation and involvement in decision making. These results reflect previous findings that women prefer to be more active in the patient–doctor relationship [21], are more focused in informational content [22], prone to discuss therapeutic interventions and preventive care [23], and thus yield consultations with better patient-centered communication [24, 25].

Our findings also suggest that older people systematically report more positive experiences, in contrast to those of younger age groups. These results are in line with those of other studies that stressed that older patients are more positive on reporting their experiences of care [3, 26–29]. This may occur because older people are more likely to use health care services on a regular basis due to chronic conditions and thus prone to develop longstanding patient–doctor relationships. Moreover, according to some studies, doctors are more likely to have patient-centered consultations with patients over age 65 [30], partly because they show more complex care needs, need to understand and interact with several sources of health information, and are more exposed to uncoordinated care [31, 32].

Our findings suggest that less educated respondents report fewer positive experiences in doctor–patient communication and involvement in decision making. This seemed to be the case even when accounted for other factors in the multivariate regression. This finding contrast to those of other studies that suggested that people with lower education are more positive towards reporting their experiences [3, 26]. On the one hand, this contradiction may partly be explained in light of findings of another study that suggested that secondary education or less was associated with doctor’s investing less time on patient’s questions, assessing their health knowledge, and negotiation [33], and thus yielding less patient-centric consultations. On the other hand, because the less educated may have poorer competencies on understanding and act upon health information, they may be limited on their ability to fully engage in meaningful patient–doctor communication and be considered a partner in decision making [34].

Also, people with paid jobs reported lesser positive experiences with the opportunity of asking questions and raising concerns. This result may suggest that people who are working, expect to interact more with the doctor, possibly to justify their efforts of overcoming time restrictions to access health care.

Our data supported that patient experiences vary significantly across type of consultation. This is in line with previous research that showed that quality of patient–doctor communication accounted for a large portion of variability on patient experience (46.6%), whereas system-level factors accounted for 27.9–47.7% [35]. Our results also stressed that the respondents have reported much better care experiences with doctors than with allied health professionals (e.g., nursing professionals), which is aligned with another study [29]. Albeit nurses affect only some dimensions of patient experience [36], one may report worse experiences if one does not receive the type of consultation expected (e.g., talk to a nurse instead of a GP) [37]. Our findings suggest that the respondents have a clear preference to be seen by a doctor, regardless of the type of care needed. It can also highlight relational problems with other health professionals that are yet to be unfold (e.g., trust, communication, confidentiality). In our study, consultations with specialist doctors in private outpatient facilities show the highest proportion of positive care experiences both with communication and involvement of the patient in decision making. These results for specialist doctors in private outpatient facilities may suggest that patients rate their experience reflecting on better accessibility to care [38], but also may seek to justify their choice for paying out-of-pocket and time invested in receiving care [3].

### Strengths and limitations

The strengths of our study lie in its representative sample and the use of a standardized set of questions to measure patient experience in Hungary that allows to establish cross-national comparisons for the first time. However, our findings should be interpreted in light of some limitations. Respondents’ characteristics might vary depending on the method of survey delivery. Because this survey was online based, non-internet users and people with low skills on information and communications technologies (e.g., the elderly) had little chance to participate. Moreover, those who were asked to take the survey but refused might have answered differently to our questions. However, we believe that this had little impact on our results, as the share of non-response (declined to answer or not sure) was below 2% for each question. Also, respondents reported their experience based on the last outpatient consultation/examination within the previous 12 months to the survey. Hence, respondents may have incurred in recall bias. Greater time-gaps between the last outpatient consultation/examination and respondents' report on the experience may have yielded reports of worst experiences, as suggested elsewhere [3, 4, 39, 40]. Furthermore, we collected patient experiences with closed questions, hence limiting respondent’s ability to provide further explanations on their answers. Notwithstanding, the use of open questions in a web-based survey would have raised other issues to the results interpretability.

### Policy relevance and future research

Our paper contributes to the Hungarian health system performance by presenting the first results on patient experiences in outpatient care, assessed with a national representative sample. Although Hungary has started to measure the performance of the health system, no routinely data are collected to know how patients perceive and experience care. Hence, it is necessary that policy-makers coordinate efforts to include patient-reported experience measures as one of the indicators to be collected system-wide. Besides giving patients the opportunity to be heard and shape the health system, it is necessary to identify proper mechanisms by which other stakeholders (e.g., providers, doctors, insurers, employers) can be involved in those movements to improve experiences of care. These actions, combined with effective monitoring and publicly reporting, can later contribute to develop policies for the improvement of Hungarians’ experiences of care.

Our findings suggested larger proportions of positive experiences on timing of consultations and doctors providing easy to understand explanations, but improvements are needed with regard to doctors giving patients opportunities to ask questions and be involved in decision making. In addition, our findings suggested that women, people under 35 years old, people with a paid job, and patients with consultations with allied health professionals reported significant less positive care experiences. These findings can be used by policy makers to further analyze factors that may explain the differences across these groups. Later, policy makers can initiate a more targeted approach by prioritizing and direct interventions aimed at enhancing better experiences of care to those groups, namely by: channeling fair incentives to providers to promote patient-centered care delivery; increase awareness to the importance of patient-reported measures and strengthen the development of soft-skills during doctors’ and nurses’ training; involve doctors’ and nurses’ professional associations in widening awareness on the subgroups with poorer experiences of care and overall cultural awareness on the importance of shared decision making to increase the value of health care outcomes.

In light of these findings, other studies could follow exploring a mixed methods approach. Our understanding of what attributes of experience of care patients value most needs to be further developed. By exploring patients’ narratives on their experiences of care, especially of those who reported less positive experience in this survey, we could gain access to richer and more detailed data on the causes. By bridging these approaches, one can better understand patients’ points of view on the health system and enhance its responsiveness to patients’ expectations and care needs.

## Electronic supplementary material

Below is the link to the electronic supplementary material.

Supplementary material 1 (PDF 188 kb)
