# Supplementary material for: Patient experiences with outpatient care in Hungary: results of an online population survey
Source: Eur J Health Econ. 2019 May 16;20(Suppl 1):79–90. doi: 10.1007/s10198-019-01064-z (PMC6544599; doi:10.1007/s10198-019-01064-z)
Supplement: Supplementary file 1 — Supplementary material 1 (PDF 188 kb) [file 10198_2019_1064_MOESM1_ESM.pdf]

Patient experiences with outpatient care in Hungary: results of an online population survey

European Journal of Health Economics (Special Issue)

Authors: Óscar Brito Fernandes[1,2] , Petra Baji[1] , Dionne Kringos[2] , Niek Klazinga[2], László Gulácsi[1] , Armin Lucevic[1,2] , Imre Boncz[3], Márta Péntek[1]

Title: Patient experiences with outpatient care in Hungary: results of an online population survey

Authors' affiliations

[1] Department of Health Economics, Corvinus University of Budapest, Fővám tér 8., H-1093 Budapest, Hungary

[2] Department of Public Health, Amsterdam UMC, University of Amsterdam, Amsterdam Public Health research institute, Meibergdreef 9, 1105 AZ Amsterdam, The Netherlands

3 Institute for Health Insurance, Faculty of Health Sciences, University of Pécs, Mária u. 5-7., H-7621 Pécs, Hungary

Corresponding author

Márta Péntek

Corvinus University of Budapest

Department of Health Economics

H-1093 Budapest, Fővám tér 8.

E-mail: marta.pentek@uni-corvinus.hu

Supplementary material

Fig. S1 Doctor spending enough time with patient in consultation, 2010, 2013 and 2016 (or nearest year)

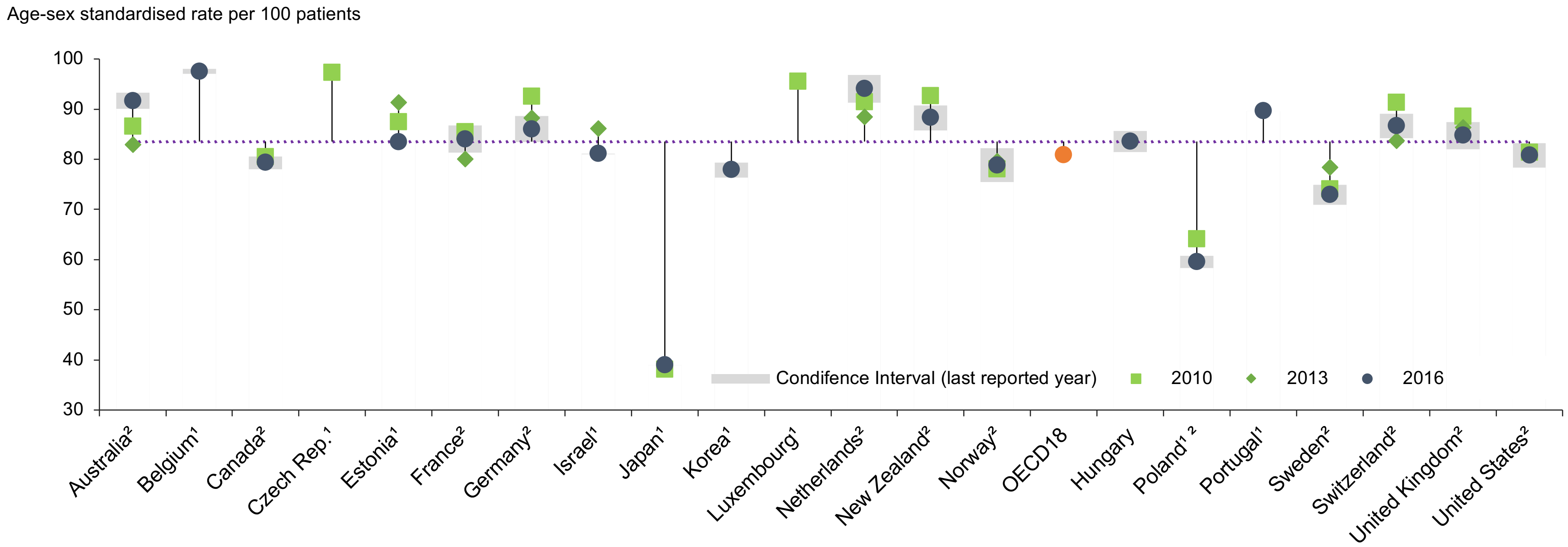

The graphic compiles data retrieved from the OECD.Stat (https://stats.oecd.org) using the health care quality indicators database. The sources of that database were the Commonwealth Fund International Health Policy Survey 2016 and other national sources.
The 95% confidence intervals have been calculated for all countries and is represented by grey areas.
Countries with superscript 1: data from national sources and; 2: data refer to patient experiences with regular doctor.
Hungarian data are not standardized against OECD 2010 population but is retrieved from a national representative sample of Hungary, adjusted for gender, age, highest education level attained, type of settlement, and region of residence.

Supplementary material

Fig. S2 Doctor providing easy-to-understand explanations, 2010, 2013 and 2016 (or nearest years)

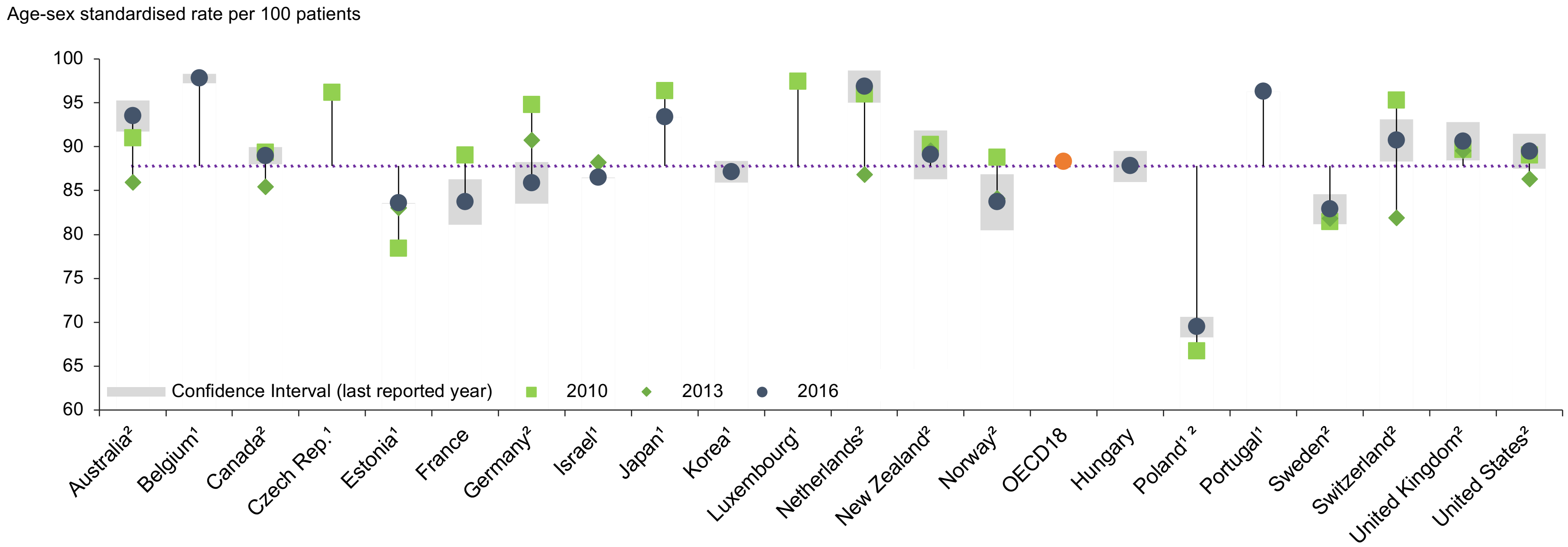

The graphic compiles data retrieved from the OECD.Stat (https://stats.oecd.org) using the health care quality indicators database. The sources of that database were the Commonwealth Fund International Health Policy Survey 2016 and other national sources.
The 95% confidence intervals have been calculated for all countries and is represented by grey areas.
Countries with superscript 1: data from national sources and; 2: data refer to patient experiences with regular doctor.
Hungarian data are not standardized against OECD 2010 population but is retrieved from a national representative sample of Hungary, adjusted for gender, age, highest education level attained, type of settlement, and region of residence.

Supplementary material

Fig. S3 Doctor giving opportunity to ask questions or raise concerns, 2010, 2013 and 2016 (or nearest years)

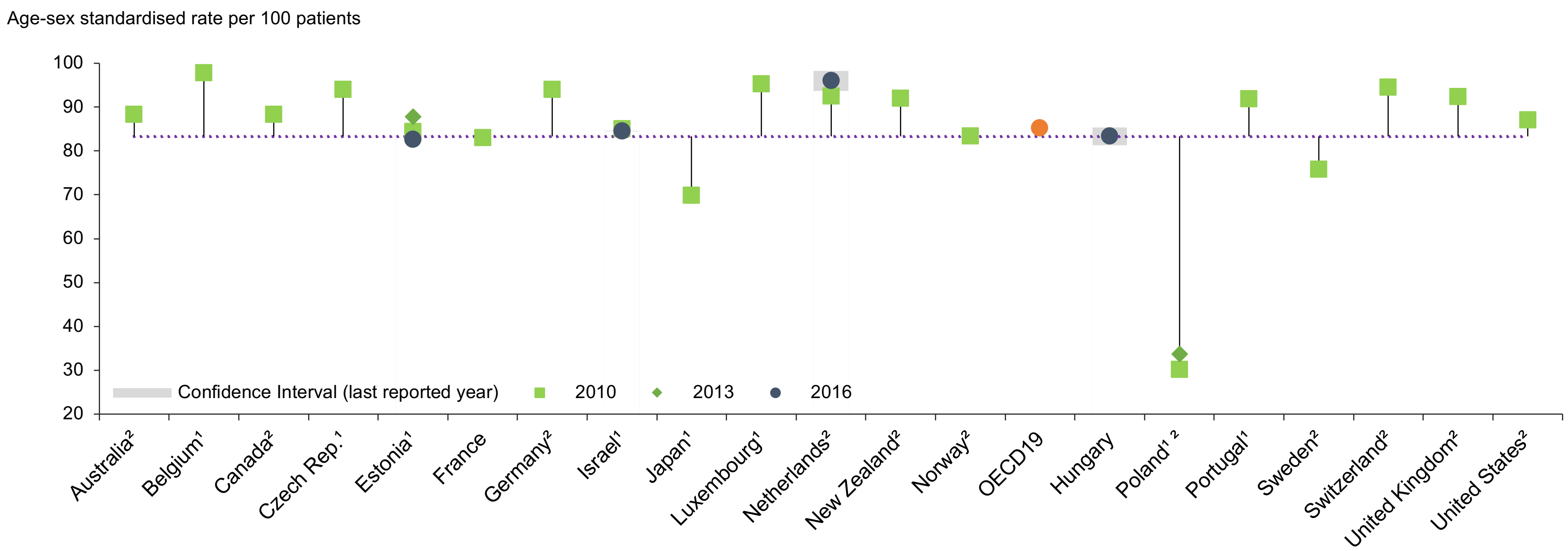

The graphic compiles data retrieved from the OECD.Stat (https://stats.oecd.org) using the health care quality indicators database. The sources of that database were the Commonwealth Fund International Health Policy Survey 2016 and other national sources.
The 95% confidence intervals have been calculated for all countries and is represented by grey areas.
Countries with superscript 1: data from national sources and; 2: data refer to patient experiences with regular doctor.
Hungarian data are not standardized against OECD 2010 population but is retrieved from a national representative sample of Hungary, adjusted for gender, age, highest education level attained, type of settlement, and region of residence.

Supplementary material

Fig. S4 Doctor involving patient in decisions about care and treatment, 2010, 2013 and 2016 (or nearest years)

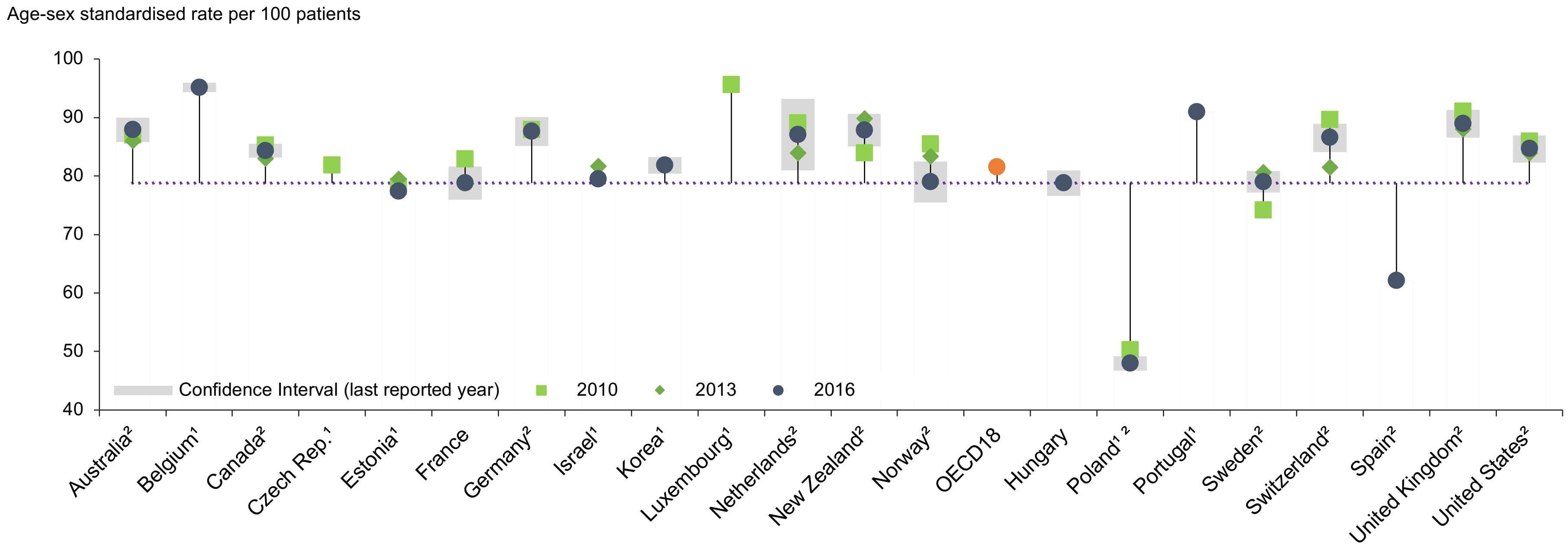

The graphic compiles data retrieved from the OECD.Stat (https://stats.oecd.org) using the health care quality indicators database. The sources of that database were the Commonwealth Fund International Health Policy Survey 2016 and other national sources.
The 95% confidence intervals have been calculated for all countries and is represented by grey areas.
Countries with superscript 1: data from national sources and; 2: data refer to patient experiences with regular doctor.
Hungarian data are not standardized against OECD 2010 population but is retrieved from a national representative sample of Hungary, adjusted for gender, age, highest education level attained, type of settlement, and region of residence.
